# The Chain Length Distribution of an Ideal Reversible Deactivation Radical Polymerization

**DOI:** 10.3390/polym10080887

**Published:** 2018-08-08

**Authors:** Simon Harrisson

**Affiliations:** Laboratoire des IMRCP, Université de Toulouse, CNRS UMR 5623, Université Paul Sabatier, 118 route de Narbonne, 31062 Toulouse CEDEX 9, France; harrisson@chimie.ups-tlse.fr; Tel.: +33-5-6155-6968

**Keywords:** polymer, reversible deactivation radical polymerization, chain length distribution, negative binomial distribution

## Abstract

The chain length distribution (CLD) of a reversible deactivation radical polymerization at full conversion is shown to be a negative binomial distribution with parameters that are simple functions of the number-average degree of polymerization and either the chain transfer constant (in the case of polymerizations that incorporate a reversible chain transfer step) or the concentrations of dormant polymer chains and deactivating agent and the rate constants of propagation and deactivation (other types of RDRP). Expressions for the CLD at intermediate conversions are also derived, and shown to be consistent with known expressions for the number-average degree of polymerization and dispersity. It is further demonstrated that these CLDs are well-approximated by negative binomial distributions with appropriate choice of parameters. The negative binomial distribution is thus a useful model for CLDs of reversible deactivation radical polymerizations.

## 1. Introduction

**Scheme 1:**
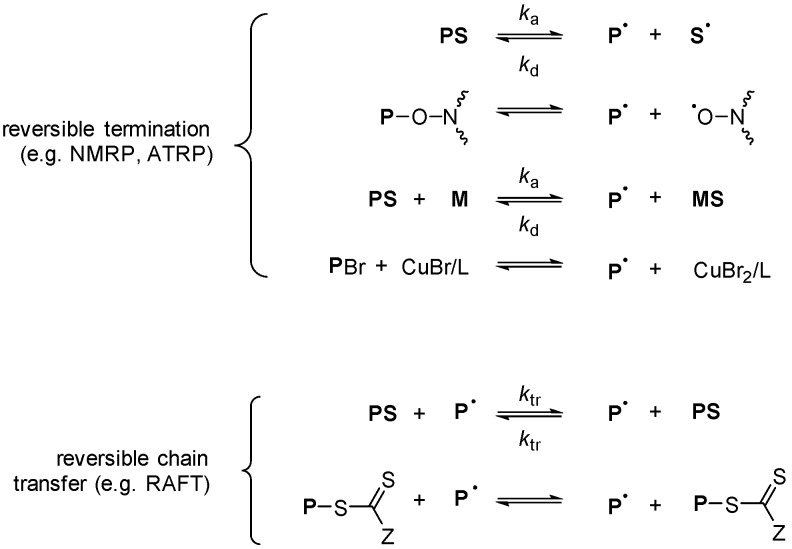
General mechanism of the reversible deactivation step in reversible deactivation radical polymerization.

Reversible deactivation radical polymerization (RDRP) encompasses a range of polymerization techniques developed over the last 25 years that permit the synthesis of well-defined, relatively narrowly-dispersed polymers via a radical process. The fundamental feature of RDRP is that dormant polymer chains are reversibly activated to form propagating radicals which can grow by adding one or more monomer units before returning to the dormant state (Scheme 1) [1].

RDRP techniques are widely used to generate highly functional materials with complex architectures (e.g., block, star, comb, etc.). In particular, several recent studies have applied RDRP to the preparation of highly complex structures containing large numbers of functional blocks [2,3,4,5,6,7,8,9].

The true precision of such complex structures is difficult to quantify. One approach is to assume near-perfect control of the polymerization, with the independent addition of monomers to each chain [10]. This is analogous to an anionic polymerization, and results in a Poisson distribution of chain lengths, with dispersity, Ð, given by Equation (1) [11].

(1) $$Ð=1+1/DP_{n}$$

In reality, monomers do not add independently of one another, as multiple monomers may be added during a single activation-deactivation cycle (with rate constant *k*_p_). Assumption of a Poisson chain length distribution (CLD) will thus lead to an underestimation of the true degree of structural variation. It would be useful to have a simple model that more accurately reflects the CLD of polymers produced by RDRP techniques.

Early work on RDRP kinetics led to the derivation of a formula for the dispersity of polymers produced by RDRP processes as a function of conversion, *c* (Equation (2)) [12,13,14].

(2) $$Ð=1+1/DP_{n}+\left(\frac{2-c}{c}\right)\left(\frac{k_{p}\left[\mathrm{PS}\right]}{k_{d}\left[S^{*}\right]}\right)$$

In Equation (2), [S*] is the concentration of deactivating agent (assumed to be constant with rate constant of deactivation *k*_d_). This could represent nitroxide radicals in nitroxide-mediated radical polymerization (NMRP, S* represented as S**˙** in Scheme 1) [15], copper(II) species in atom transfer radical polymerization (ATRP, S* represented as MS in Scheme 1) [16], or reversible addition-fragmentation chain transfer agents (RAFT agents) in RAFT polymerization (in this case S* and PS are identical) [17].

In many forms of RDRP, the deactivating agent is derived from the dormant polymer (e.g., an alkyl halide in ATRP or alkoxyamine in NMRP), designated as PS in Equation (2) and Scheme 1. In reversible chain transfer polymerizations such as RAFT polymerization, the deactivating agent and the dormant polymer are identical. If the ratio of [S*] to [PS] is assumed to be constant (equal to 1 for reversible chain transfer polymerizations), an apparent chain transfer constant, *C*_S_, can be defined (Equation (3)).

(3) $$C_{S}=\frac{k_{d}\left[S^{*}\right]}{k_{p}\left[\mathrm{PS}\right]}$$

Substitution into Equation (2) gives Equation (4) [12,13].

(4) $$Ð=1+1/DP_{n}+\left(\frac{2-c}{c}\right)\cdot 1/C_{S}$$

This simple formula has been extended by Zhu et al. to account for side reactions such as irreversible termination, which cause broadening of the CLD as the reaction proceeds to high conversion [18].

While numerous expressions exist for the dispersity under more or less realistic assumptions, there are relatively few examples of expressions for the entire CLD [19]. Gold proposed a modified Poisson distribution which takes into account unequal initiation and propagation rates, but assumes the independent addition of monomers [20]. Muller et al. proposed an expression for the CLD of an RDRP process with slow equilibration between active and dormant species, but assumed a constant monomer concentration [21]. Tobita [22,23] has derived an expression for the CLD of an RDRP continuous stirred tank reactor (constant monomer concentration) process as a hypergeometric function combining the Poisson and geometric (most probable) distributions. Finally, Konkolewicz et al. have developed a recursive equation for the CLD of a batch RDRP by convoluting Poisson and geometric distributions while taking into account the changing monomer concentration in the batch process [24,25,26]. The resulting CLD is given by Equation (5) [24].

(5) $$\mathrm{Pr}\left(DP=k\right)=\overset{\infty }{\underset{j=0}{\sum }}\frac{\mu_{\mathrm{decap}}^{j}e^{-\mu_{\mathrm{decap}}}}{j!}\mathrm{Pr}\left(DP=k|N=j\right)$$

In Equation (5), *μ*_decap_ is the mean number of activation cycles that the chains have undergone, while Pr(*DP* = *k*|*N* = *j*) represents the probability that a chain that has been activated *j* times has a degree of polymerization of *k*. This probability is given by the recursive formula of Equation (6).

(6) $$\begin{array}{c} \mathrm{Pr}\left(DP=k|N=j\right)=\overset{m}{\underset{i=0}{\sum }}\mathrm{Pr}\left(DP=k-i|N=j-1\right)\frac{{\left[M\right]}_{0}}{C_{S}\left[S^{*}\right]}{\left(1-\frac{1}{C_{S}}\right)}^{j-1}\\ \mathrm{Pr}\left(DP=k|N=0\right)=\{\begin{array}{ll} 1, & k=0\\ 0, & k>0 \end{array} \end{array}$$

In this article, a similar approach to that of Tobita and Konkolewicz et al. is used to show that the CLD of a polymer produced by an ideal RDRP batch process at full conversion is given by the negative binomial distribution with parameters *C*_S_ and *DP*_n_/(*DP*_n_ + *C*_S_) (Equation (7)).

(7) $$\mathrm{Pr}\left(DP=k\right)=\frac{\Gamma \left(k+C_{S}\right)}{k!\Gamma \left(C_{S}\right)}{\left(\frac{C_{S}}{DP_{n}+C_{S}}\right)}^{C_{S}}{\left(\frac{DP_{n}}{DP_{n}+C_{S}}\right)}^{k}$$

The distribution at intermediate conversions is also derived, as well as parameters for a negative binomial distribution that closely approximates the true distribution. First derived in 1714 [27], the negative binomial distribution is one of the fundamental distributions of statistics, and is frequently used to model discrete data whose variance is greater than their mean [28]. The considerable body of statistical literature on this distribution could thus be applied to the study of polymer CLDs.

## 2. Materials and Methods

All simulations were carried out using Excel 2013 software on a desktop computer. The code used to generate simulated distributions of 10,000 chains is given in the Supporting Information.

## 3. Results

We define an ideal RDRP as one in which all rate constants are independent of chain length and conversion, and no termination or other side reactions take place.

The rate of propagation is given by Equation (8).

(8) $$R_{p}=-\frac{d\left[M\right]}{dt}=k_{p}\left[M\right]\left[P^{*}\right]$$

In this equation, [M] represents the monomer concentration, and [P*], the concentration of active chains (assumed to be constant).

In terms of conversion, *c*:

(9) $$R_{p}={\left[M\right]}_{0}\frac{dc}{dt}=k_{p}{\left[M\right]}_{0}\left(1-c\right)\left[P^{*}\right]$$

(10) $$c=1-e^{-k_{p}\left[P^{*}\right]t}$$

Chain deactivation occurs at a constant rate, *R*_d_, given by Equation (11).

(11) $$R_{d}=k_{d}\left[S^{*}\right]\left[P^{*}\right]$$

The probability, *p*, that an active chain will propagate is given by

(12) $$p=\frac{R_{p}}{R_{p}+R_{d}}=\frac{k_{p}\left[M\right]}{k_{p}\left[M\right]+k_{d}\left[S^{*}\right]}=\frac{\left[M\right]/\left[\mathrm{PS}\right]}{\left[M\right]/\left[\mathrm{PS}\right]+k_{d}\left[S^{*}\right]/k_{p}\left[\mathrm{PS}\right]}$$

Defining *T* as the target degree of polymerization at full conversion, equal to [M]_0_/[PS], we have

(13) $$p=\frac{T\left(1-c\right)}{T\left(1-c\right)+C_{S}}$$

In a single activation-deactivation cycle, an active chain will add *n* monomers, where *n* is a geometrically distributed random variable [22,23,24,25,26]. The probability that *k* monomers are added in a single activation/deactivation cycle at time *t* is given by

(14) $$\mathrm{Pr}{\left(n=k\right)}_{t}=p^{k}\left(1-p\right)$$

As the concentration of monomer changes during the polymerization, the instantaneous distribution of *n* also varies. The total distribution of *n*, representing all activation/deactivation cycles from the beginning of the polymerization (*t* = 0) to a time *t* corresponding to conversion *c*, is obtained by integrating with respect to time:

(15) $$\mathrm{Pr}\left(n=k\right)=\frac{\int_{0}^{t}{\left(\frac{T\left(1-c\right)}{T\left(1-c\right)+C_{S}}\right)}^{k}\left(\frac{C_{S}}{T\left(1-c\right)+C_{S}}\right)dt}{\int_{0}^{t}dt}$$

In terms of conversion, this is

(16) $$\mathrm{Pr}\left(n=k\right)=\frac{\int_{0}^{c}{\left(\frac{T\left(1-c\right)}{T\left(1-c\right)+C_{S}}\right)}^{k}\left(\frac{C_{S}}{T\left(1-c\right)+C_{S}}\right)\frac{dc}{1-c}}{-\ln\left(1-c\right)}$$

Evaluation of the integral gives

(17) $$\mathrm{Pr}\left(n=k\right)=\{\begin{array}{c} 1+\frac{\ln\left(\frac{1-p_{f}}{1-p_{0}}\right)}{\ln\left(1-c\right)},k=0\\ \frac{{\left(p_{0}\right)}^{k}-{\left(p_{f}\right)}^{k}}{-k\ln\left(1-c\right)},k\geq 1 \end{array}$$

where

$$p_{0}=\frac{T}{T+C_{S}},\;p_{f}=\frac{T\left(1-c\right)}{T\left(1-c\right)+C_{S}}$$

This distribution has a probability generating function [29], *G*_n_(*z*), given by Equation (18):

(18) $$G_{n}\left(z\right)=\overset{\infty }{\underset{k=0}{\sum }}z^{k}\mathrm{Pr}\left(n=k\right)=1+\frac{\ln\left[\frac{\left(1-p_{0}z\right)\left(1-p_{f}\right)}{\left(1-p_{0}\right)\left(1-p_{f}z\right)}\right]}{\ln\left(1-c\right)}$$

Throughout the polymerization, activation/deactivation cycles occur at a constant rate. The number, *N*, of activation/deactivation cycles experienced by each chain is Poisson distributed, with an expected number of cycles per chain equal to the total number of cycles divided by the total number of chains [22,23,24,25,26].

(19) $$E\left(N\right)=\frac{k_{d}\left[S^{*}\right]\left[P^{*}\right]t}{\left[\mathrm{PS}\right]}=-C_{S}\ln\left(1-c\right)$$

The corresponding probability generating function *G_N_*(*z*) is given by Equation (20):

(20) $$G_{N}\left(z\right)=e^{-C_{S}\ln\left(1-c\right)\left(z-1\right)}$$

Each chain consists of *N* segments of length *n*_1_, *n*_2_, …, *n_N_*, where *n_i_* are independent, identically distributed random variables with probability generating function *G*_n_(*z*) and *N* is Poisson-distributed with expected value of −*C*_S_ln(1 − *c*).

The distribution of total chain lengths, *Y* = *n*_1_ + *n*_2_ + … + *n_N_*, is thus a compound Poisson distribution with probability generating function *G_Y_*(*z*) given by Equation (21):

(21) $$G_{Y}\left(z\right)=G_{N}\left(G_{n}\left(z\right)\right)={\left[\frac{\left(1-p_{0}\right)\left(1-p_{f}z\right)}{\left(1-p_{0}z\right)\left(1-p_{f}\right)}\right]}^{C_{S}}$$

The probability mass function of the CLD can be obtained from the derivatives of *G_Y_*(*z*) evaluated at *z* = 0, leading to the recursive formula of Equation (22):

(22) $$\mathrm{Pr}\left(Y=k\right)=\frac{G_{Y}^{\left(k\right)}\left(0\right)}{k!}=\{\begin{array}{c} {\left(\frac{1-p_{0}}{1-p_{f}}\right)}^{C_{S}}={\left[\frac{T\left(1-c\right)+C_{S}}{T+C_{S}}\right]}^{C_{S}},\;k=0\\ \frac{C_{S}}{k}\overset{k}{\underset{i=1}{\sum }}\left({\left(p_{0}\right)}^{i}-{\left(p_{f}\right)}^{i}\right)\;\mathrm{Pr}\left(Y=k-i\right),\;k\geq 1 \end{array}$$

**Figure 1:**
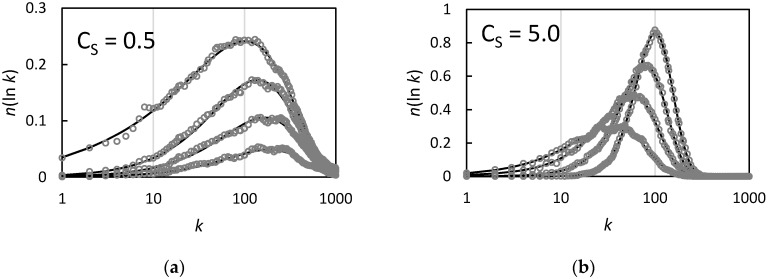
Comparison of calculated CLDs (solid lines) with simulated distributions (open circles, 10,000 chains) for polymerizations with *T* = 100, *C*_S_ = 0.5 (**a**) or 5 (**b**), conversion = 0.25, 0.5, 0.75 and 1.

Figure 1 shows the comparison of calculated CLDs obtained from Equation (22) and simulated CLDs for *C*_S_ of 0.5 and 5 and conversions of 25–100%. Additional comparisons of calculated and simulated CLDs for a wider range of chain transfer constants may be found in the Supporting Information (Figures S1–S4).

The expected value and dispersity of the CLD can also be obtained from *G_Y_*(*z*), and give

(23) $$DP_{n}=\underset{z\to 1^{-}}{\lim}G_{Y}^{\prime }\left(z\right)=cT$$

(24) $$Ð=\underset{z\to 1^{-}}{\lim}\left(\frac{G_{Y}^{\prime \prime }\left(z\right)+G_{Y}^{\prime }\left(z\right)}{{\left[G_{Y}^{\prime }\left(z\right)\right]}^{2}}\right)=1+\frac{1}{DP_{n}}+\left(\frac{2-c}{c}\right)\cdot 1/C_{S}$$

Equation (24) is identical to the expression derived by Müller et al. [13] (Equation (4)) using the method of moments [30].

The above expressions include chains of length 0, which would not normally be included in the determination of molecular weight. Corrected values for *DP*_n_ and Ð can be obtained by excluding chains of zero length [13] to give:

(25) $$DP_{n}^{k\neq 0}=\frac{cT}{\left(1-{\left[\frac{T\left(1-c\right)+C_{S}}{T+C_{S}}\right]}^{C_{S}}\right)}$$

(26) $${Ð}^{k\neq 0}=\left(1+\frac{1}{cT}+\frac{2-c}{c.C_{S}}\right)\left(1-{\left[\frac{T\left(1-c\right)+C_{S}}{T+C_{S}}\right]}^{C_{S}}\right)$$

As the conversion approaches 1, *p*_f_ approaches 0, and *G_Y_*(*z*) approaches:

(27) $$\underset{X\to 1}{\lim}G_{Y}\left(z\right)={\left[\frac{\left(1-p_{0}\right)}{\left(1-p_{0}z\right)}\right]}^{C_{S}}$$

This is the probability generating function of a negative binomial distribution with parameters *C*_S_ and *p*_0_. Thus, the CLD of a polymer prepared under ideal RDRP batch conditions at full conversion is a negative binomial distribution, with probability mass function given by Equation (7), number average chain length equal to *T*, and dispersity given by Equation (28).

(28) $$Ð=1+1/DP_{n}+1/C_{S}$$

## 4. Discussion

While the negative binomial distribution strictly describes the CLD only at full conversion, it also provides a good approximation to CLDs at intermediate conversions. The full CLD (including zero-length chains) is approximated by the random variable *Z* with probability mass function given by Equation (29), where *Z* is a mixture of a negative binomial distribution with parameters *ρ* and *π* and a constant random variable representing unreacted chains:

(29) $$\begin{array}{c} \mathrm{Pr}\left(Z=k\right)=\{\begin{array}{c} {\left(1-c\right)}^{C_{S}}+\left(1-{\left(1-c\right)}^{C_{S}}\right){\left(1-\pi \right)}^{\rho },\;k=0\\ \left(1-{\left(1-c\right)}^{C_{S}}\right)\left(\begin{array}{c} k+\rho -1\\ k \end{array}\right){\left(1-\pi \right)}^{\rho }\pi^{k},\;k\geq 1 \end{array}\\ \rho ={\left(\left(1+\frac{2-c}{c.C_{S}}\right)\left(1-{\left(1-c\right)}^{C_{S}}\right)-1\right)}^{-1}\\ \pi =1-{\left(1+cT+\frac{T\left(2-c\right)}{C_{S}}-\frac{cT}{1-{\left(1-c\right)}^{C_{S}}}\right)}^{-1} \end{array}$$

**Figure 2:**
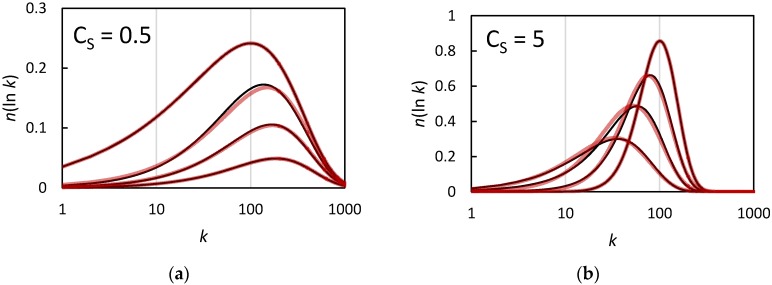
Comparison of negative binomial approximation (thick red lines) with calculated CLDs (thin black lines) for polymerizations with *T* = 100, *C*_S_ = 0.5 (**a**) or 5 (**b**), conversion = 0.25, 0.5, 0.75 and 1.

As shown in Figure 2, the approximation is best for low conversions (when the CLD is approximately geometric), high conversions (when the CLD is approximately negative binomial) and *C*_S_ close to 1 (when the CLD is approximately geometric) or ≳50 (when the CLD is approximately Poisson). A comparison between approximate and calculated distributions for a wide range of *C*_S_ and *T* is given in the Supporting Information (Figures S5–S8). The quality of the approximation is comparable to that of the commonly-used [31,32,33,34] Schulz-Zimm distribution [35,36] (Equation (30)).

(30) $$\begin{array}{c} n\left(k\right)=\frac{k^{z-1}y^{z}e^{-yk}}{\Gamma \left(z\right)}\\ z=\frac{1}{Ð-1},\;y=\frac{z}{DP_{n}} \end{array}$$

Comparisons of the negative binomial and Schulz-Zimm distributions are presented in the Supporting Information, Table S1, Figures S9–S12.

The results obtained in this work assume an initial degree of polymerization of 0, and are thus valid for chains grown from a small molecule initiator or chain transfer agent, or individual blocks of a block copolymer. The total CLD of a block copolymer is given by the sum of the CLDs of its individual blocks. This sum is not a negative binomial distribution unless all blocks were polymerized to full conversion and *DP*_n_/(*DP*_n_ + *C*_S_) is the same for each block. In real polymerizations, the presence of side reactions such as termination and irreversible chain transfer may result in significant deviations from the ideal case considered here [18]. However, when relatively short chains are targeted, such reactions may be disregarded [6,7]. Thus the negative binomial distribution may usefully serve as a model for the CLD of short blocks within multiblock copolymers prepared by RDRP [4,5,6,7,8,9]. Additionally, the simplicity of the negative binomial distribution and its implementation in readily-available software such as Excel™ facilitates the visualization of CLDs as an aid to teaching or comprehension. This allows features such as the number of unreacted chains or the relative proportions of high and low molecular weight polymer to be estimated, and provides a more nuanced picture of the overall CLD than the dispersity alone.

## 5. Conclusions

The chain length distribution of an ideal RDRP at full conversion is given by a negative binomial distribution with parameters *C*_S_ and *DP*_n_/(*DP*_n_ + *C*_S_). Unlike previously derived expressions for the CLD of an RDRP process, this distribution can be expressed as a simple function of two fundamental parameters of the polymerization. This simplicity is achieved, however, at the cost of assumptions such as the complete absence of termination, which will not hold in real polymerizations. Nevertheless, such a simple model will be useful in situations where side-reactions may be assumed to be negligible, and complements more complex models such as those of Tobita [22,23] and Konkolewicz et al. [24,25,26] that deal with more realistic systems that incorporate irreversible termination and transfer reactions.

## Supplementary Materials

The following are available online at http://www.mdpi.com/2073-4360/10/8/887/s1. Simulated CLDs for a wide range of *C*_S_ and targeted degrees of polymerization, accompanied by calculated CLDs and negative binomial approximations. Comparison of goodness of fit of negative binomial approximation and Schulz-Zimm distribution to calculated CLDs. Details of procedure used to simulate the CLD.
