# Supplementary material for: The Chain Length Distribution of an Ideal Reversible Deactivation Radical Polymerization
Source: Polymers (Basel). 2018 Aug 8;10(8):887. doi: 10.3390/polym10080887 (PMC6403729; doi:10.3390/polym10080887)
Supplement: Supplementary file 1 [file polymers-10-00887-s001.pdf]

Supporting Information for 'The chain length distribution of an ideal reversible deactivation radical polymerization'

Simon Harrisson

Laboratoire des IMRCP, Université de Toulouse, CNRS UMR 5623, Université Paul Sabatier, 118 route de Narbonne 31062 Toulouse Cedex 9, France

Figures S1-S4: Comparison of simulated and calculated CLDs

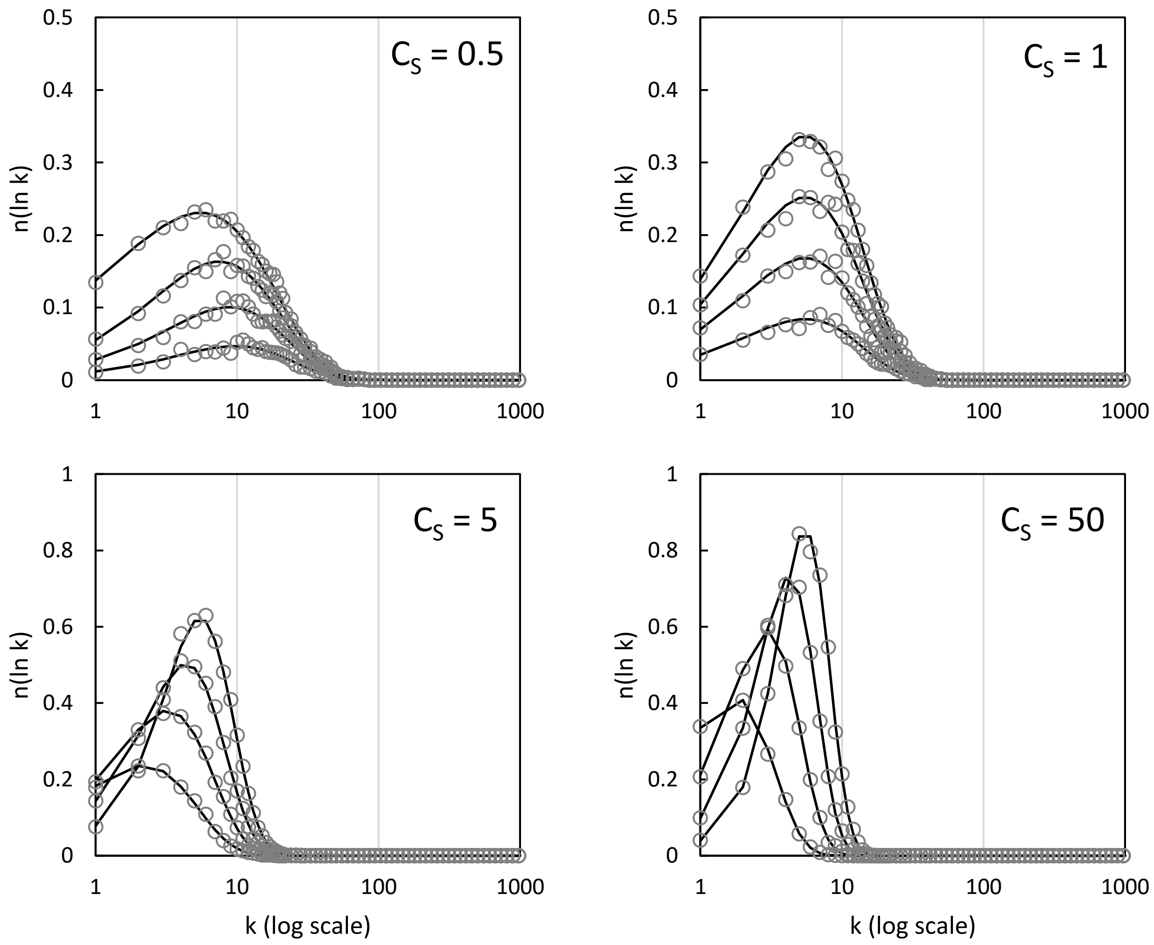

Figure S1. Comparison of simulated (circles) and calculated CLDs (lines) for targeted DP_n = 5, C_S = 0.5, 1, 5, and 50, conversion = 0.25, 0.5, 0.75, and 1

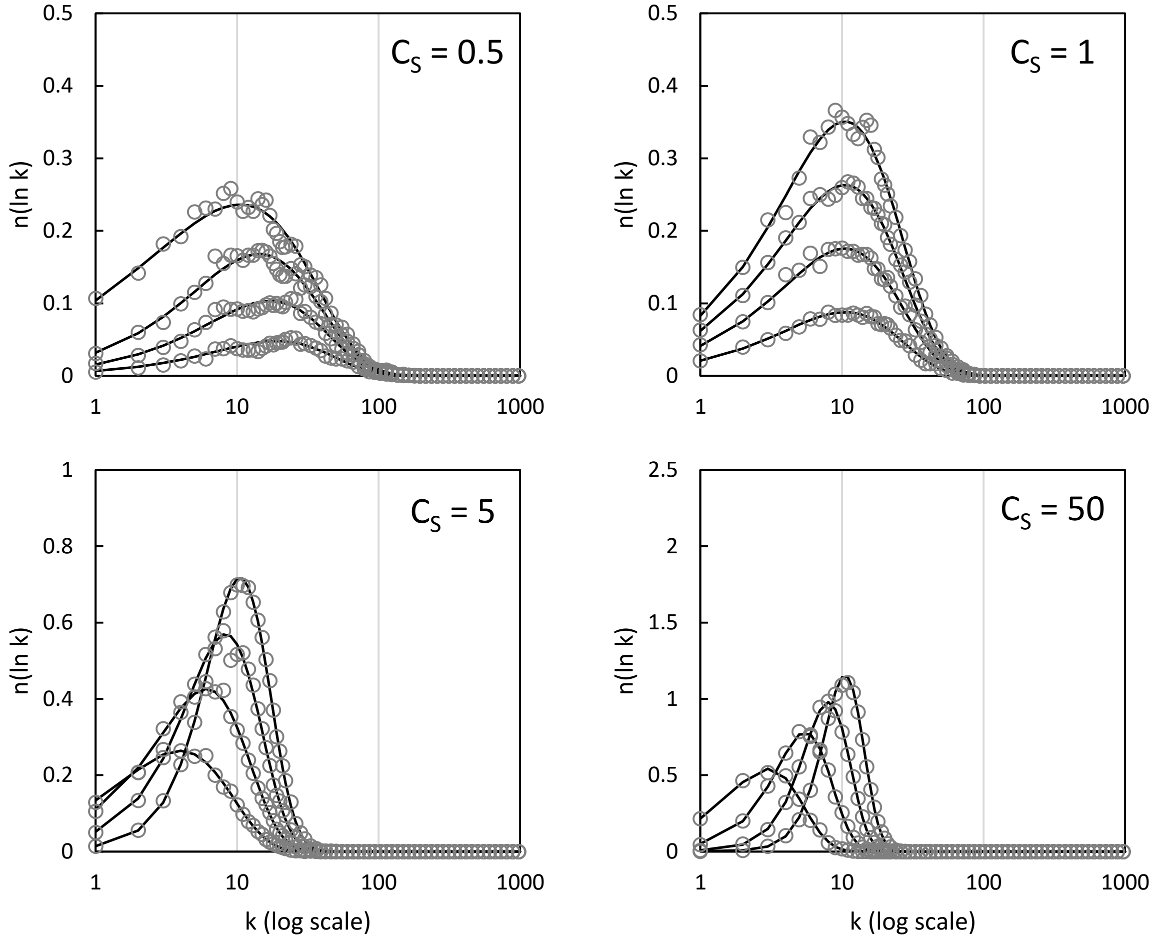

Figure S2. Comparison of simulated (circles) and calculated CLDs (lines) for targeted DP_n = 10, C_S = 0.5, 1, 5, and 50, conversion = 0.25, 0.5, 0.75, and 1

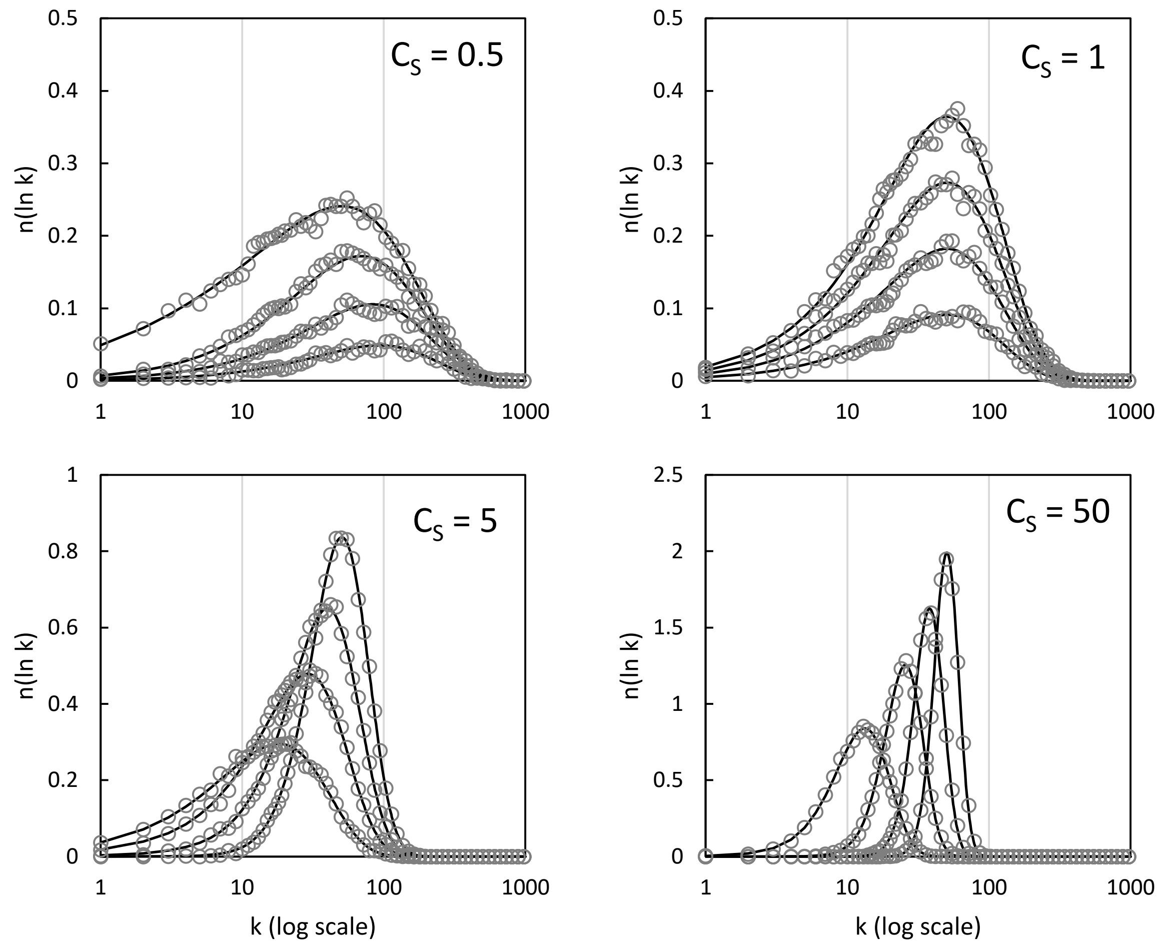

Figure S3. Comparison of simulated (circles) and calculated CLDs (lines) for targeted DP_n = 50, C_S = 0.5, 1, 5, and 50, conversion = 0.25, 0.5, 0.75, and 1

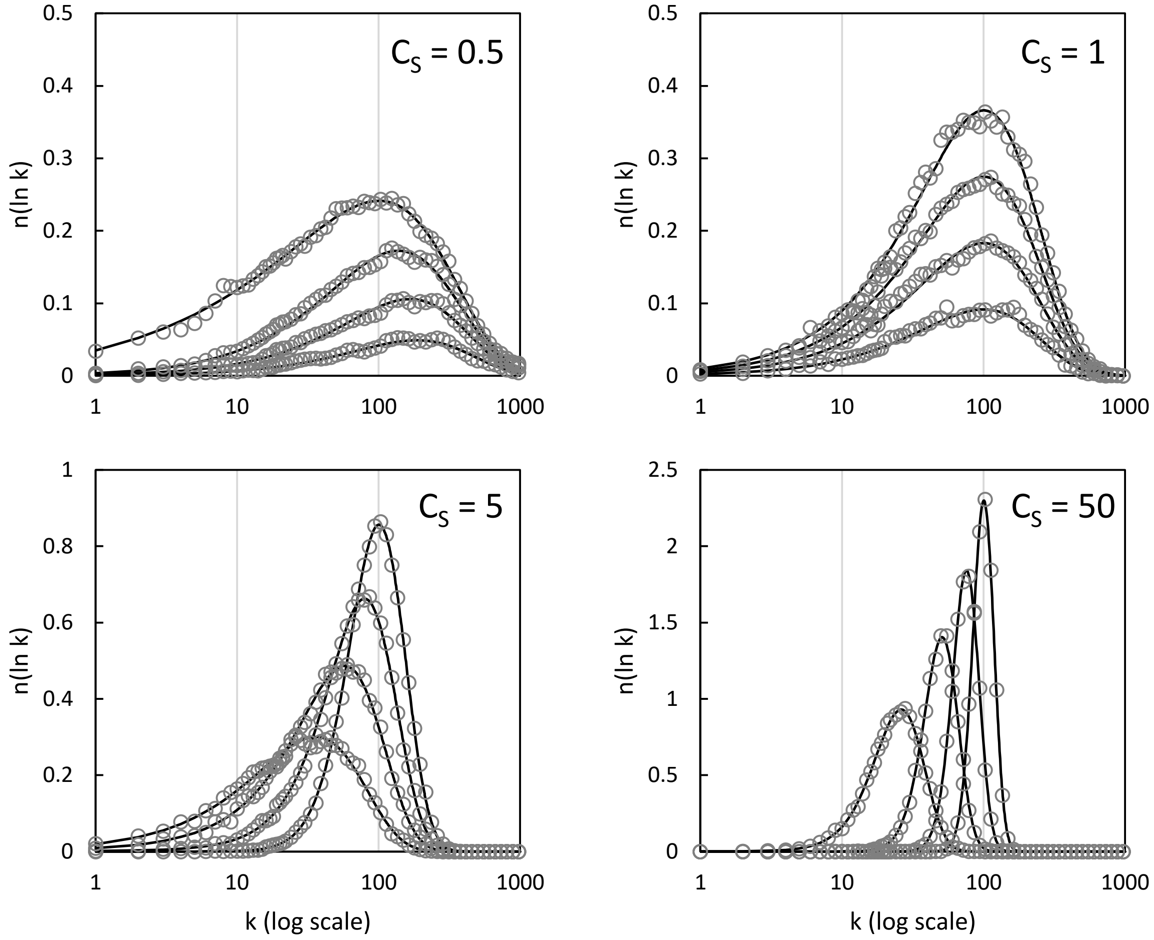

Figure S4. Comparison of simulated (circles) and calculated CLDs (lines) for targeted DP_n = 100, C_S = 0.5, 1, 5, and 50, conversion = 0.25, 0.5, 0.75, and 1

Figures S5-S8. Comparison of calculated CLDs and negative binomial approximations

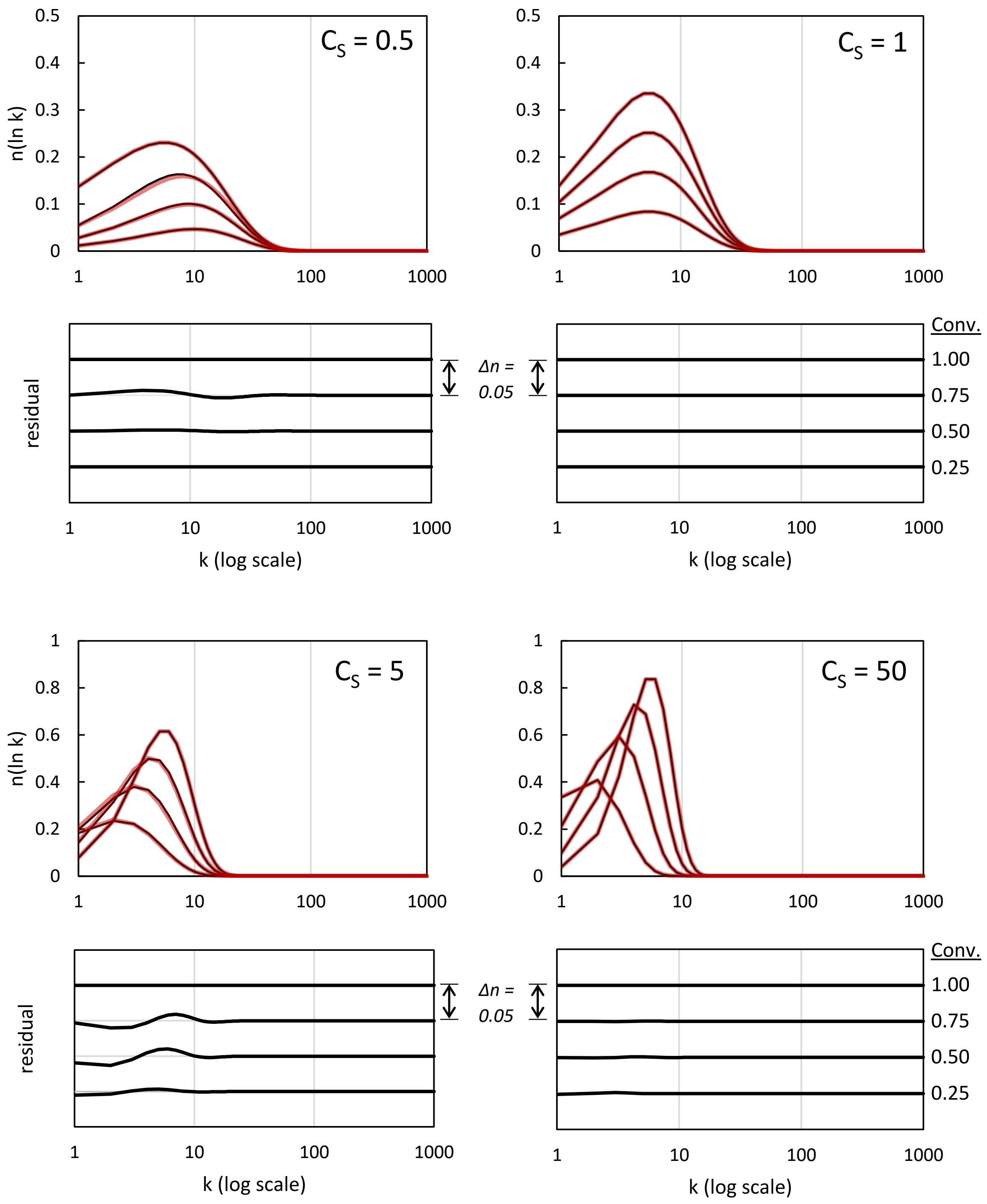

Figure S5. Comparison of calculated CLD (black) and negative binomial approximation (red). Target DP_n = 5, C_S = 0.5, 1, 5, and 50, conversion = 0.25, 0.5, 0.75, and 1. Lower charts show difference between calculated CLD and approximation as a function of chain length, *k*.

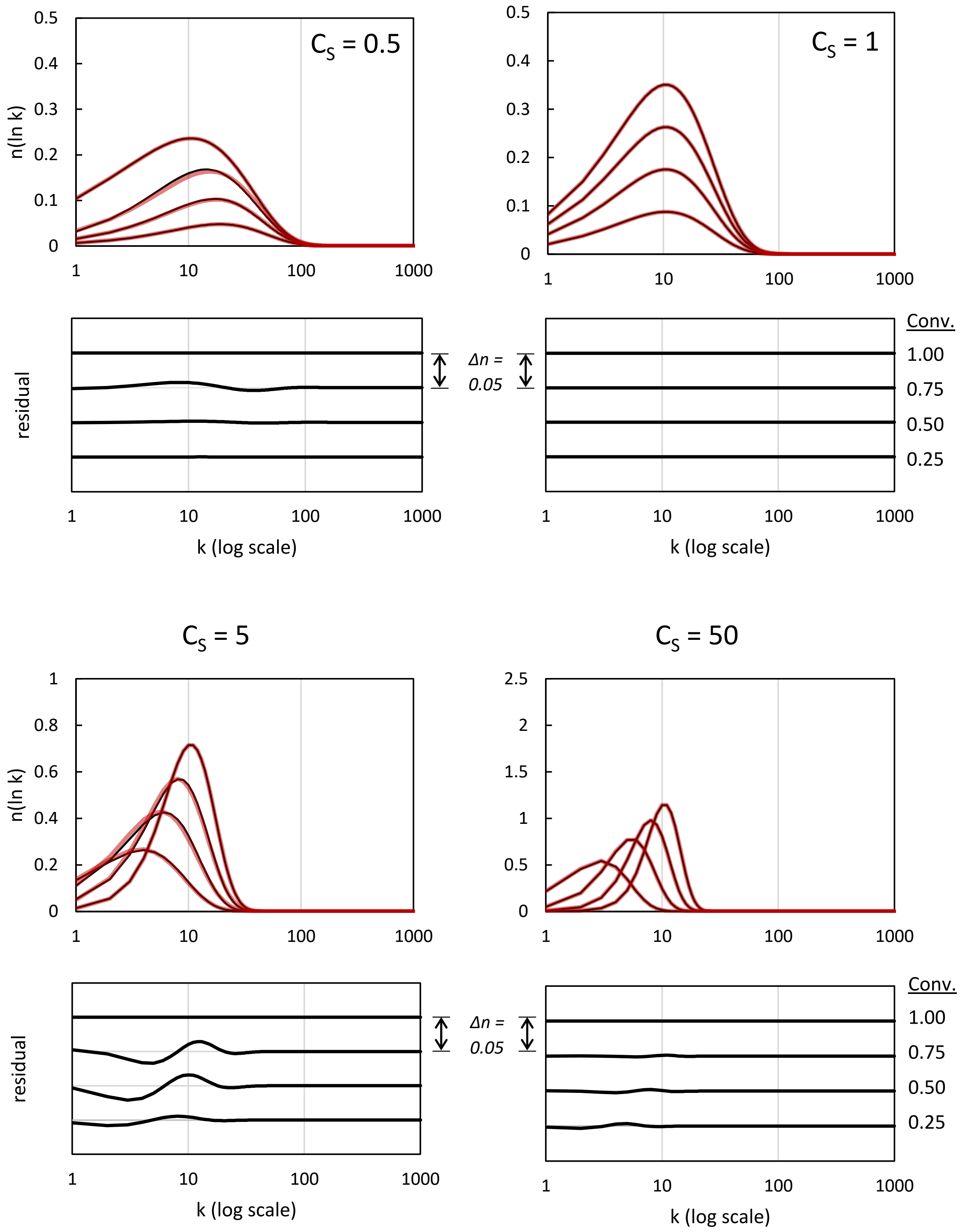

Figure S6. Comparison of calculated CLD (black) and negative binomial approximation (red). Target DP_n = 10, C_S = 0.5, 1, 5, and 50, conversion = 0.25, 0.5, 0.75, and 1. Lower charts show difference between calculated CLD and approximation as a function of chain length, *k*.

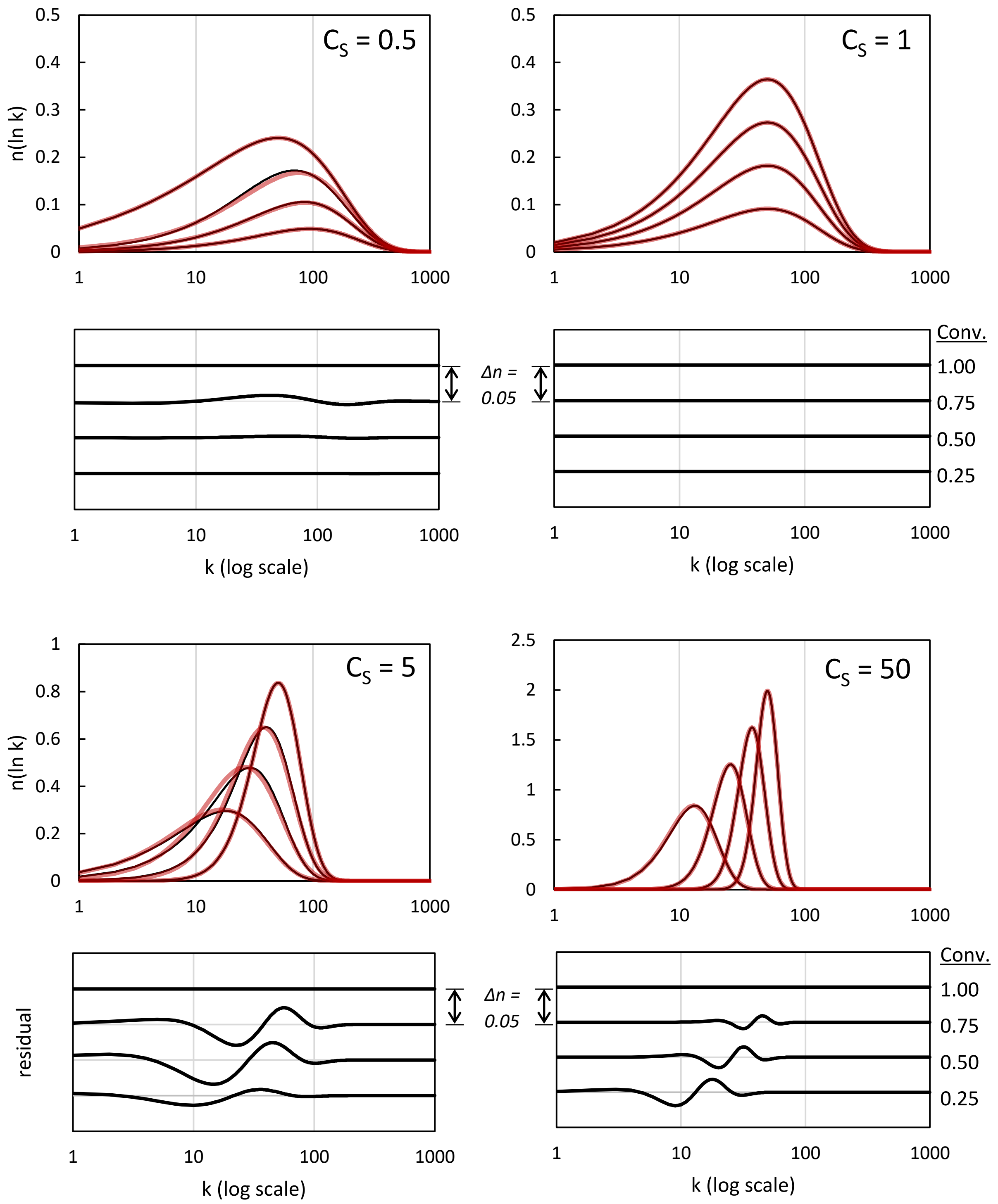

Figure S7. Comparison of calculated CLD (black) and negative binomial approximation (red). Target DP_n = 50, C_S = 0.5, 1, 5, and 50, conversion = 0.25, 0.5, 0.75, and 1. Lower charts show difference between calculated CLD and approximation as a function of chain length, *k*.

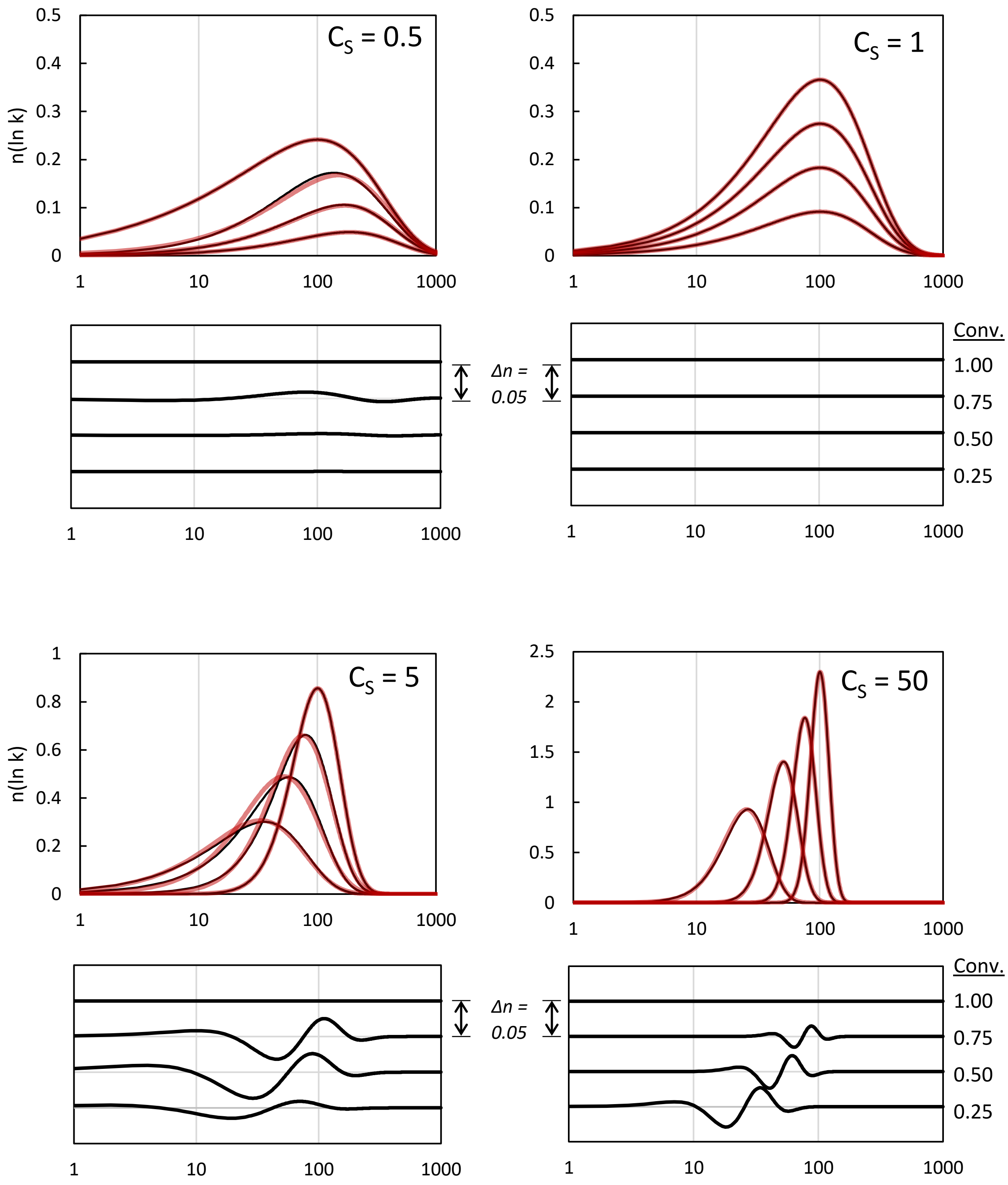

Figure S8. Comparison of calculated CLD (black) and negative binomial approximation (red). Target DP_n = 100, C_S = 0.5, 1, 5, and 50, conversion = 0.25, 0.5, 0.75, and 1. Lower charts show difference between calculated CLD and approximation as a function of chain length, *k*.

Schulz-Zimm distribution

The Schulz-Zimm distribution (Schulz, G. V. *Z. Physik. Chem.* **1939**, *B43*, 25; Zimm, B. H. *J. Chem. Phys.* **1948**, *16*, 1099) is given by equation S1.

$$n(k) = \frac{k^{z-1} y^{z} e^{-yk}}{\Gamma(z)} \tag{S1}$$

In this equation, *k* is the chain length, and *z* and *y* are adjustable parameters that are related to the dispersity, Đ, and the number-average degree of polymerization, DP_n, by equations S2 and S3:

$$z = \frac{1}{Đ - 1} \tag{S2}$$

$$y = \frac{z}{DP_n} \tag{S3}$$

Table S1. Parameters of Schulz-Zimm distribution for Target DP of 5, 10, 50 and 100, C_S of 0.5, 1, 5 and 50, and conversion of 0.25, 0.5, 0.75 and 1.

Target DP	C_S	conversion	$DP_{n,k\neq0}$[a]	$Đ_{k\neq0}$[b]	z[c]	y[d]
5	0.5	0.25	10.33	1.911	1.097	0.106
		0.5	9.562	1.935	1.070	0.112
		0.75	8.602	2.005	0.995	0.116
		1	7.158	2.235	0.810	0.113
5	1	0.25	6.000	1.833	1.200	0.200
		0.5	6.000	1.833	1.200	0.200
		0.75	6.000	1.833	1.200	0.200
		1	6.000	1.833	1.200	0.200
5	5	0.25	2.566	1.559	1.789	0.697
		0.5	3.278	1.525	1.903	0.581
		0.75	4.145	1.447	2.235	0.539
		1	5.161	1.356	2.807	0.544
5	50	0.25	1.830	1.325	3.073	1.680
		0.5	2.771	1.317	3.151	1.137
		0.75	3.863	1.262	3.818	0.988
		1	5.043	1.210	4.771	0.946
10	0.5	0.25	19.67	1.958	1.044	0.0531
		0.5	18.10	1.989	1.011	0.0559
		0.75	16.11	2.079	0.927	0.0575
		1	12.79	2.424	0.702	0.0549
10	1	0.25	11.00	1.909	1.100	0.100
		0.5	11.00	1.909	1.100	0.100
		0.75	11.00	1.909	1.100	0.100
		1	11.00	1.909	1.100	0.100
10	5	0.25	4.180	1.675	1.420	0.355
		0.5	5.758	1.563	1.776	0.308
		0.75	7.742	1.421	2.376	0.307
		1	10.04	1.295	3.394	0.338
10	50	0.25	2.838	1.357	2.804	0.988
		0.5	5.065	1.244	4.103	0.810
		0.75	7.509	1.165	6.053	0.806
		1	10.00	1.120	8.342	0.834

Target DP	C_S	conversion	$DP_{n,k\neq0}$[a]	$Đ_{k\neq0}$[b]	z[c]	y[d]
50	0.5	0.25	94.31	1.999	1.001	0.0106
		0.5	86.39	2.037	0.964	0.0112
		0.75	76.12	2.148	0.871	0.0114
		1	55.52	2.719	0.582	0.0105
50	1	0.25	51.00	1.980	1.020	0.0200
		0.5	51.00	1.980	1.020	0.0200
		0.75	51.00	1.980	1.020	0.0200
		1	51.00	1.980	1.020	0.0200
50	5	0.25	17.25	1.797	1.255	0.0727
		0.5	26.27	1.561	1.783	0.0679
		0.75	37.62	1.356	2.812	0.0748
		1	50.00	1.220	4.546	0.0909
50	50	0.25	12.52	1.218	4.577	0.366
		0.5	25.00	1.010	10.00	0.400
		0.75	37.50	1.060	16.67	0.444
		1	50.00	1.040	25.00	0.500
100	0.5	0.25	187.6	2.004	0.996	0.00531
		0.5	171.7	2.044	0.958	0.00558
		0.75	151.1	2.157	0.864	0.00572
		1	107.6	2.798	0.556	0.00517
100	1	0.25	101	1.990	1.010	0.0100
		0.5	101	1.990	1.010	0.0100
		0.75	101	1.990	1.010	0.0100
		1	101	1.990	1.010	0.0100
100	5	0.25	33.64	1.814	1.229	0.0365
		0.5	52.05	1.556	1.798	0.0345
		0.75	75.14	1.344	2.906	0.0387
		1	100.0	1.210	4.762	0.0476
100	50	0.25	25.00	1.180	5.560	0.222
		0.5	50.00	1.080	12.50	0.250
		0.75	75.00	1.047	21.43	0.286
		1	100.0	1.030	33.33	0.333

[a] DP_n excluding zero-length chains. Calculated from equation 23 of main text. [b] Dispersity excluding zero-length chains. Calculated from equation 24 of main text. [c] Calculated from equation S2. [d] Calculated from equation S3.

Figures S9 to S12. Comparison of negative binomial and Schulz-Zimm approximations

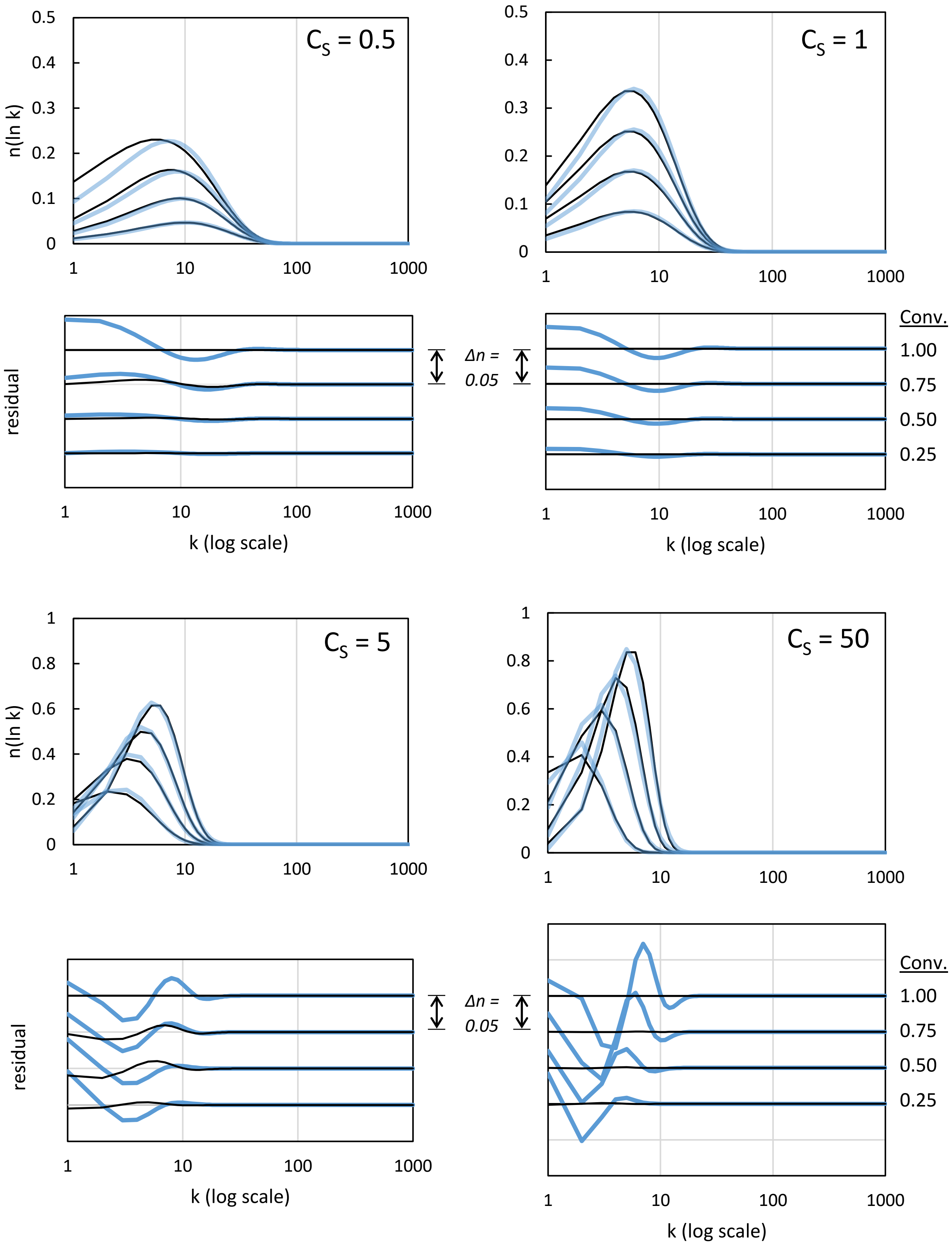

Figure S9. Comparison of calculated CLD (black) and Schulz-Zimm approximation (blue). Target DP_n = 5, C_S = 0.5, 1, 5, and 50, conversion = 0.25, 0.5, 0.75, and 1. Lower charts show difference between calculated CLD and Schulz-Zimm (blue) or negative binomial (black) approximations as a function of chain length, *k*.

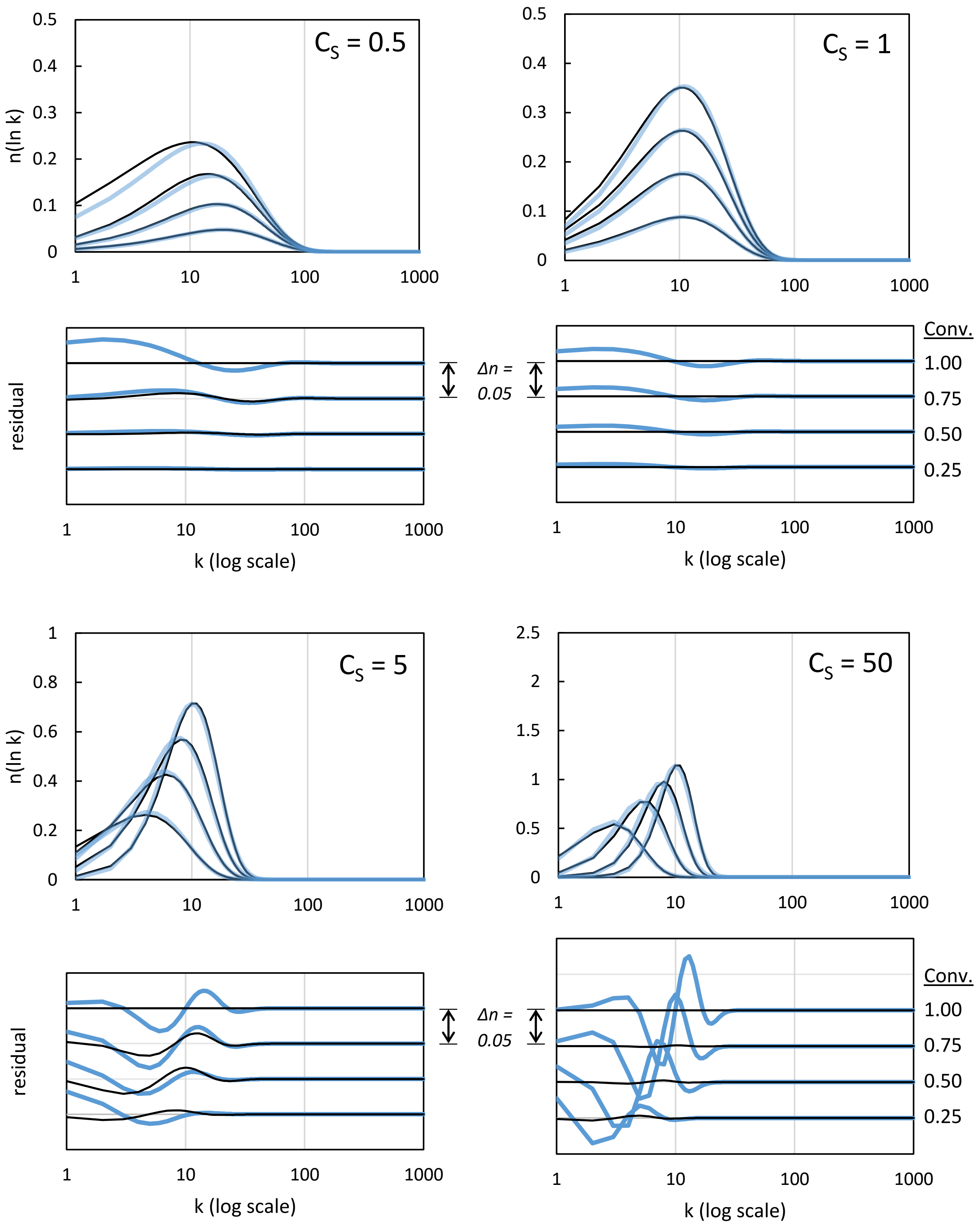

Figure S10. Comparison of calculated CLD (black) and Schulz-Zimm approximation (blue). Target DP_n = 10, C_S = 0.5, 1, 5, and 50, conversion = 0.25, 0.5, 0.75, and 1. Lower charts show difference between calculated CLD and Schulz-Zimm (blue) or negative binomial (black) approximations as a function of chain length, *k*.

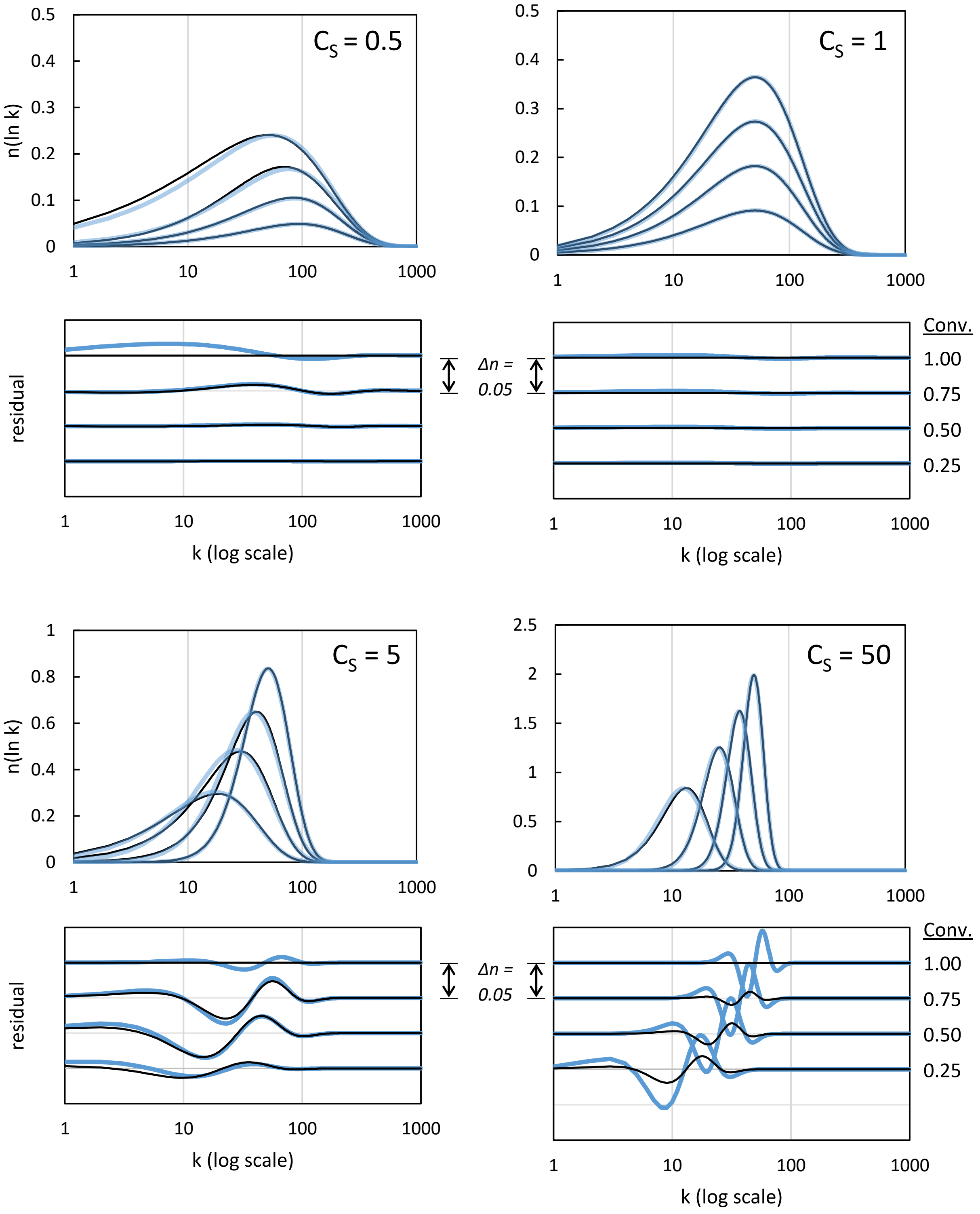

Figure S11. Comparison of calculated CLD (black) and Schulz-Zimm approximation (blue). Target DP_n = 50, C_S = 0.5, 1, 5, and 50, conversion = 0.25, 0.5, 0.75, and 1. Lower charts show difference between calculated CLD and Schulz-Zimm (blue) or negative binomial (black) approximations as a function of chain length, *k*.

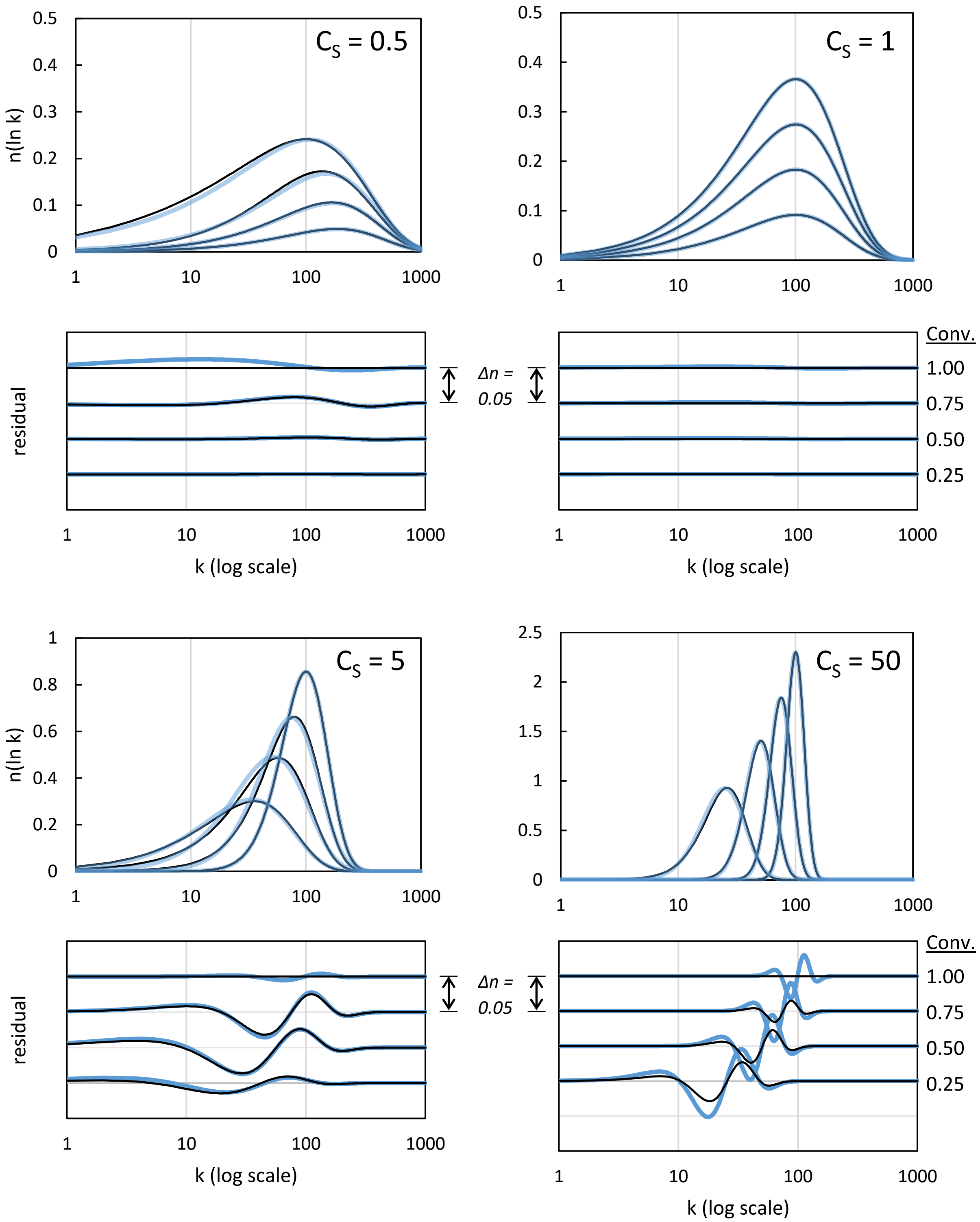

Figure S12. Comparison of calculated CLD (black) and Schulz-Zimm approximation (blue). Target DP_n = 100, C_S = 0.5, 1, 5, and 50, conversion = 0.25, 0.5, 0.75, and 1. Lower charts show difference between calculated CLD and Schulz-Zimm (blue) or negative binomial (black) approximations as a function of chain length, *k*.

Details of simulation of CLD

Chains were simulated using an Excel spreadsheet containing the following macro:

```
Private Sub ChainGrowthLiving()
Dim i As Integer
Dim j As Integer
Dim Chain() As Integer
Dim PropCount As Long
Dim MaxPropCount As Long
Dim Transfer As Boolean
Dim Conversion As Single
Dim DelP As Single
Dim ChainLength As Long
Dim ChainCount As Long
Dim CS As Single
Dim ReportInterval As Single
Dim ConversionFloor As Single
Dim n As Integer

ChainLength = Cells(1, 6).Value 'reads target chain length (cell F1)
ChainCount = Int(Cells(2, 6).Value) - 1 'reads no. of chains (F2)
ReportInterval = 0.25 'conversion interval to output chain lengths
ConversionFloor = ReportInterval
n = 1
MaxPropCount = ChainLength * (ChainCount + 1)
ReDim Chain(0 To ChainCount) As Integer

CS = Cells(1, 8).Value 'Reads CS from cell H1
Conversion = 0
Cells(4, 1) = "Chain"
Application.ScreenUpdating = False
For j = 0 To ChainCount
    Chain(j) = 0 'erase all strings
    Cells(5 + j, 1) = j + 1
Next j
PropCount = 0
Do While PropCount < MaxPropCount
'randomly select a chain
    i = Int((ChainCount + 1) * Rnd())
    Transfer = False
    Do Until Transfer = True
        PropCount = PropCount + 1
        Chain(i) = Chain(i) + 1 'adds a monomer to the chain
        Transfer = Rnd() < CS / (ChainLength * (1 - Conversion) +
CS) 'check if chain transfer has taken place
    Loop 'until chain transfer occurs
    DelP = PropCount / MaxPropCount - Conversion
    Conversion = PropCount / MaxPropCount 'updates conversion
    If Conversion >= ConversionFloor Then 'output chain lengths
    Application.ScreenUpdating = False
        Cells(4, n + 1) = Conversion
        For j = 0 To ChainCount
            If Chain(j) = 0 Then Cells(j + 5, n + 1) = "" Else
Cells(j + 5, n + 1) = Chain(j)
        Next j
```

```
        ConversionFloor = ConversionFloor + ReportInterval 'set next
reporting threshold
        n = n + 1
    Application.ScreenUpdating = True
    End If
Loop 'until all monomer is consumed

Erase Chain
End Sub
```

Ten thousand chains were simulated for each combination of C_S (0.5, 1, 5 and 50) and target chain length (5, 10, 50 and 100) investigated.

An example of the resulting chain length distribution is shown in Figure S13 for C_S of 5, target chain length of 50, and conversions of 25%, 50%, 75% and 100%.

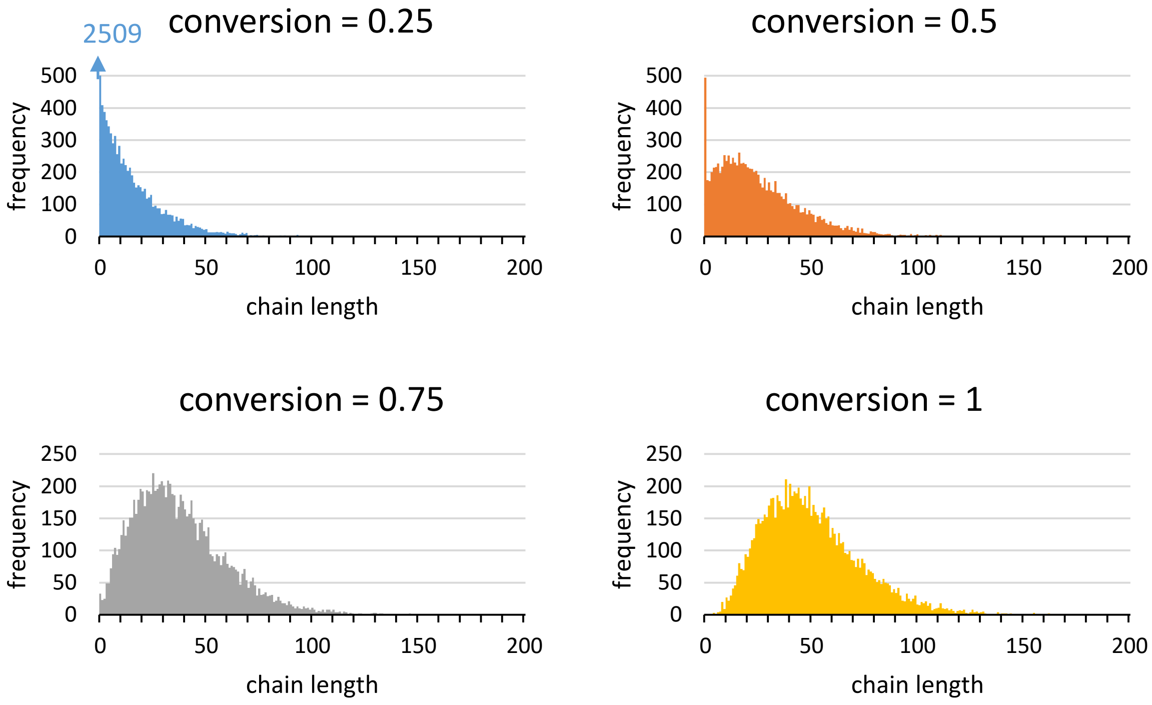

Figure S13. Chain length histograms from simulated polymerization with C_S = 5, target chain length of 50, and conversions of 0.25, 0.5, 0.75 and 1. Total number of chains = 10,000.

Simulated chain length distributions as a function of ln(chain length) were obtained from these histograms by first multiplying the observed frequency of each chain by its chain length, *k*, then smoothing using an (2.Int(0.1*k*)+1)-point moving average, where Int(*x*) is the integer part of *x* (Figure S14).

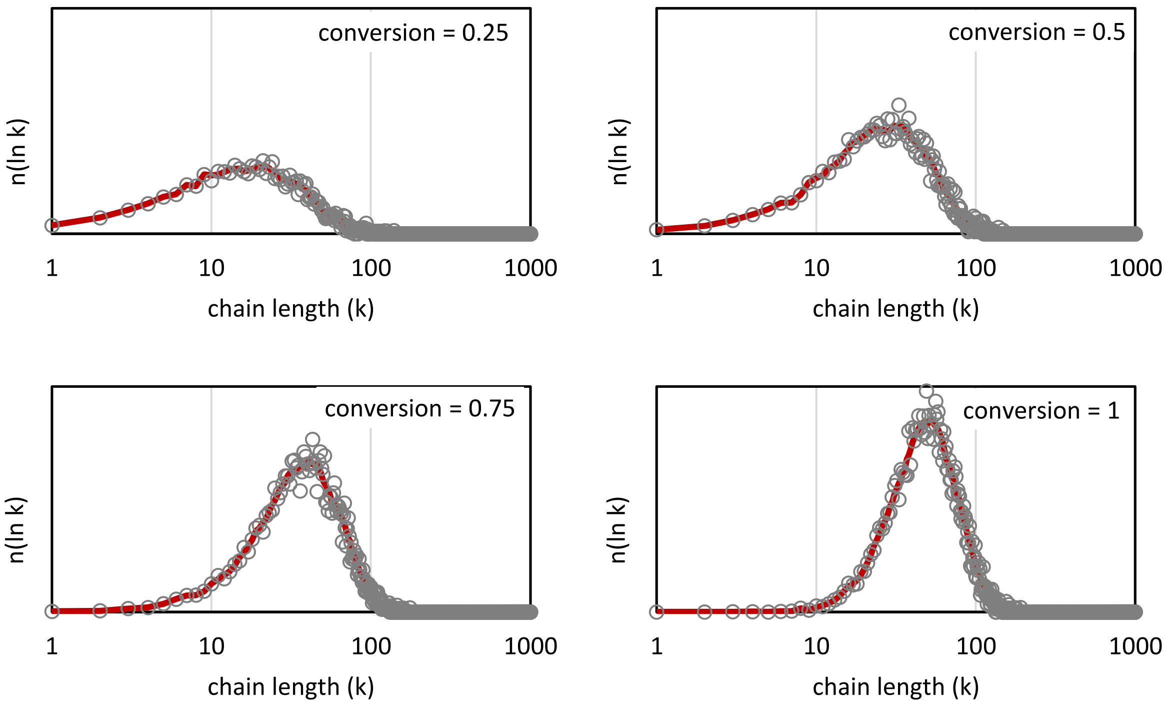

Figure S14. Chain length distributions converted to logarithmic scale ($n(\ln k) = k.n(k)$). Open circles represent raw frequency data from simulated polymerizations with C_S = 5, target chain length of 50, and conversions of 0.25, 0.5, 0.75, and 1. Red line represents frequency data after smoothing using a (2.Int(0.1*k*) + 1)-point moving average
